# Innate immune system activation in zebrafish and cellular models of Diamond Blackfan Anemia

**DOI:** 10.1038/s41598-018-23561-6

**Published:** 2018-03-26

**Authors:** Nadia Danilova, Mark Wilkes, Elena Bibikova, Min-Young Youn, Kathleen M. Sakamoto, Shuo Lin

**Affiliations:** 1 Department of Molecular, Cell & Developmental Biology, University of California, Los Angeles, CA USA; 2 Department of Pediatrics Stanford University School of Medicine, Stanford, CA USA

## Abstract

Deficiency of ribosomal proteins (RPs) leads to Diamond Blackfan Anemia (DBA) associated with anemia, congenital defects, and cancer. While p53 activation is responsible for many features of DBA, the role of immune system is less defined. The Innate immune system can be activated by endogenous nucleic acids from non-processed pre-rRNAs, DNA damage, and apoptosis that occurs in DBA. Recognition by toll like receptors (TLRs) and Mda5-like sensors induces interferons (IFNs) and inflammation. Dying cells can also activate complement system. Therefore we analyzed the status of these pathways in RP-deficient zebrafish and found upregulation of interferon, inflammatory cytokines and mediators, and complement. We also found upregulation of receptors signaling to IFNs including Mda5, Tlr3, and Tlr9. TGFb family member activin was also upregulated in RP-deficient zebrafish and in RPS19-deficient human cells, which include a lymphoid cell line from a DBA patient, and fetal liver cells and K562 cells transduced with RPS19 shRNA. Treatment of RP-deficient zebrafish with a TLR3 inhibitor decreased IFNs activation, acute phase response, and apoptosis and improved their hematopoiesis and morphology. Inhibitors of complement and activin also had beneficial effects. Our studies suggest that innate immune system contributes to the phenotype of RPS19-deficient zebrafish and human cells.

## Introduction

Diamond-Blackfan Anemia (DBA) is a bone marrow failure syndrome, which is also characterized by congenital malformations and cancer^1,2^. DBA is caused by mutations in ribosomal proteins (RPs), most often in RPS19, while mutations in several other RPs are found at lower frequencies^3,4^. The RPs affected in DBA are required for processing of pre-rRNA; their deficiency leads to the accumulation of non-processed pre-rRNA and the impairment of ribosome biogenesis^5–8^.

p53 activation is a common response to RP deficiency^9–13^. Inhibition of p53 decreases hematopoietic and developmental defects in animal models of DBA suggesting that p53 upregulation is involved in the pathogenesis of DBA. Activation of p53 independent signaling pathways in DBA has also been reported^12,14^ however their role and interaction with the p53 network is not well defined.

The role of immune system in DBA is not clear. Lymphoid cells have been suggested to play a role in DBA pathophysiology but further studies failed to demonstrate significant impact of these cells^15^. Recent analysis of the immune status of patients with various bone marrow failure conditions performed by Giri *et al*.^16^ show that most DBA patients have normal number and function of immune cells. However some data suggest upregulation of interferon (IFN) signaling and inflammation in DBA. The analysis of erythrocyte cytoplasmic proteome from DBA patients cells show increased presence of IFN targets and immunoproteasome components^17^. Increased expression of genes associated with IFN and TNF pathways has been noted in hematopoietic progenitors^18^. Importantly, interferon regulatory factor 9 (IRF9) essential for type I IFN signaling was 2.84 fold upregulated in multipotential hematopoietic progenitors. Altered expression of genes associated with inflammation, NFkB and TNF signaling have been found in fibroblasts from DBA patients such as ~14 fold increase in TNF alpha induced protein 3 (TNFAIP3)^19^. Monocytes from DBA patients are oversensitive to LPS stimulation and produce higher levels of cytokines such as TNF, IL6, and others in response to low dose of LPS^20^. The authors concluded that these patients might have “a chronic subclinical inflammatory micro-environment in the bone marrow”. TNF upregulation has also been noted in human hematopoietic progenitors with RPS19 knockdown^21^.

On the other hand, some patients with ribosomopathies demonstrate features of immunodeficiency^22,23^. A decreased proliferation rate and translational capacity of lymphoid cells from DBA patients has been reported^24^. Therefore, it is important to understand the precise contribution of not only adaptive but also innate immunity in DBA.

One of the major arms of innate immunity is complement system, a cascade of enzymes and other factors that promotes phagocytosis and inflammation and induces lysis of pathogens and compromised cells^25,26^. The central event in this cascade is a cleavage of complement factor 3 with production of a reactive C3b fragment that can covalently bind to surfaces of microbes and cells. Over activation of complement is associated with several hematologic disorders such as paroxysmal nocturnal hemoglobinuria, atypical hemolytic uremic syndrome, cold agglutinin disease and others^27^. The role of complement in DBA has not been studied.

Innate immunity includes autonomous immune mechanisms that are active not only in immune cells but also practically in all nucleated cells, especially in those of epithelial origin^28,29^. Several hundreds of proteins are involved in autonomous immune protection. They include receptors that recognize molecules specific for pathogens, called pattern recognition receptors (PRRs) and various effectors that restrict the spread of pathogen or induce cell death. In addition, infected cells produce cytokines such as interferons that activate autonomous immune mechanisms in neighboring cells. Interferons regulate a large fraction of autonomous immune genes^30^. IFN type I and III are induced when PRRs bind products from pathogens activating IFN regulatory factors (IRF) 3, 5, and 7, c-Jun, and NFkB. This leads to the formation of a signaling complex that contains STAT1 and 2 and interferon regulatory factor 9 (IRF9). This complex drives the expression of IFN-stimulated genes (ISGs).

Another innate immune mechanism is inflammation, which is associated with the recruitment of immune cells to the site of infection or injury and induction of fever. It translates autonomous response at the cellular level to the response on the level of organism. Many cells respond to infection, stress and other homeostasis-altering processes by assembly of inflammasomes^31^. These protein complexes cleave precursors of cytokines such as IL-1 and IL-18 leading to their secretion and induction of inflammation.

Innate immunity can sense not only features of pathogens but also abnormal or misplaced self-molecules^32^. Several classes of immune receptors can be activated by endogenous ligands. Mda5 (melanoma differentiation associated protein 5), an RNA helicase from Rig1-like receptor (RLR) family, recognizes dsRNA. dsRNA is not restricted to viruses; 3-UTRs of mRNAs and some non-coding RNAs such as rRNA often contain self-complementary regions that can form double-stranded structures^33,34^. Mda5 also recognizes mRNAs that lack 2-O-methylation at their 5 caps. Human studies show that MDA5 overactivation contributes to autoimmune diseases such as systemic lupus erythematosus and diabetes^34^. Toll-like receptor 3 (TLR3) recognizes dsRNA and other RNAs^35–37^. DNA sensors such as TLR9 can recognize DNA from apoptotic cells and mitochondrial DNA^37^. DNA damage is associated with the production of DNA fragments that are used in DNA repair; some of these fragments can leak into cytoplasm and activate DNA sensors^38,39^. Alternatively, RNA Pol III can convert them into dsRNA, which then is recognized by dsRNA sensors such as Mda5^40^. Abnormal composition of membranes may affect the interaction of cells with the complement system, an intrinsically unstable cascade of enzymes that can induce cell lysis and inflammation^32^. Activation of PRRs can lead to upregulation of interferons, NFkB, and inflammation. Complement system can be activated by components of apoptotic and necrotic cells^41^.

RP-deficient cells have many alterations that can activate innate immune response: they accumulate non-processed pre-rRNA^4–8^, have altered membranes^42^, have increased DNA damage^43^, and are prone to apoptosis^44^. We therefore hypothesized that DBA may be associated with increased interferon production, inflammation, and activated complement. The advantage of using zebrafish to study the role of innate immunity in DBA is the absence of a functional adaptive immune system during the first several days of zebrafish development. At this stage, the autonomous immune mechanisms, innate immune cells, and complement provide the immune protection. Therefore, contribution of these immune mechanisms to the RP-deficient phenotype can be studied without interference from T and B cells. We used a combination of two different models of RP deficiency, a genetic mutant for *rpl11* gene from a large ribosomal subunit and RP deficiency created by morpholino for *rps19* gene from a small ribosomal subunit. Using two models from different subunits and created by different mechanisms let us to discern general features of the innate immune system response to RP deficiency.

We report in this paper that interferon network was upregulated in RP-deficient zebrafish model of DBA. We found increased expression of interferon regulators and interferon-stimulated genes (ISGs) both in zebrafish Rpl11 mutant and Rps19 morphants. Genes encoding for Mda5, Tlr3, and Tlr9 receptors that signal to IFNs were upregulated. We also found upregulation of inflammatory pathways including increased expression of genes for Tnf and IL-6 (interleukin 6). Changes in expression of activin/inhibin subunits in Rps19-deficient zebrafish and RPS19-deficient human primary cells and cell lines pointed to activin upregulation. Complement system was also upregulated in RP-deficient zebrafish. Inhibitors of TLR3, activin, and complement improved condition of Rps19-deficient zebrafish. Our data suggest that the innate immune system activation could contribute to the pathophysiology of DBA.

## Results

### Zebrafish models of DBA

To study the innate immune responses in RP-deficient zebrafish, we used *rps19* gene from a small ribosomal subunit and *rpl11* gene from a large ribosomal subunit. Rpl11 mutant was generated in Nancy Hopkins lab^45^ and characterized in our lab^12^. Previously we created an Rps19-deficient fish using a morpholino, which was highly specific as was confirmed by using an alternative translational morpholino, rescue of morphant phenotype by *rps19* mRNA, and use of scrambled morpholino that had no effect on embryos at any dose studied up to 13 ng per embryo^9^. Although Rps19 mutant is also available, the morpholino model is preferable to genetic mutants in certain settings, such as when evaluating the effects of drug treatment. Rpl11 and Rps19 mutants are viable only as heterozygotes; correspondingly, mutants comprise only 25% of their progeny and to evaluate the effect of drug treatment, each embryo needs to be genotyped. In addition, morpholino-injected embryos can be analyzed at any time point during development while mutants can be reliably separated from their wild-type siblings only at 48 hpf. We also used this morpholino to create Rps19 deficiency on p53 negative background in p53 zebrafish mutant.

### Interferon network is upregulated in RP-deficient zebrafish

**Figure 1:**
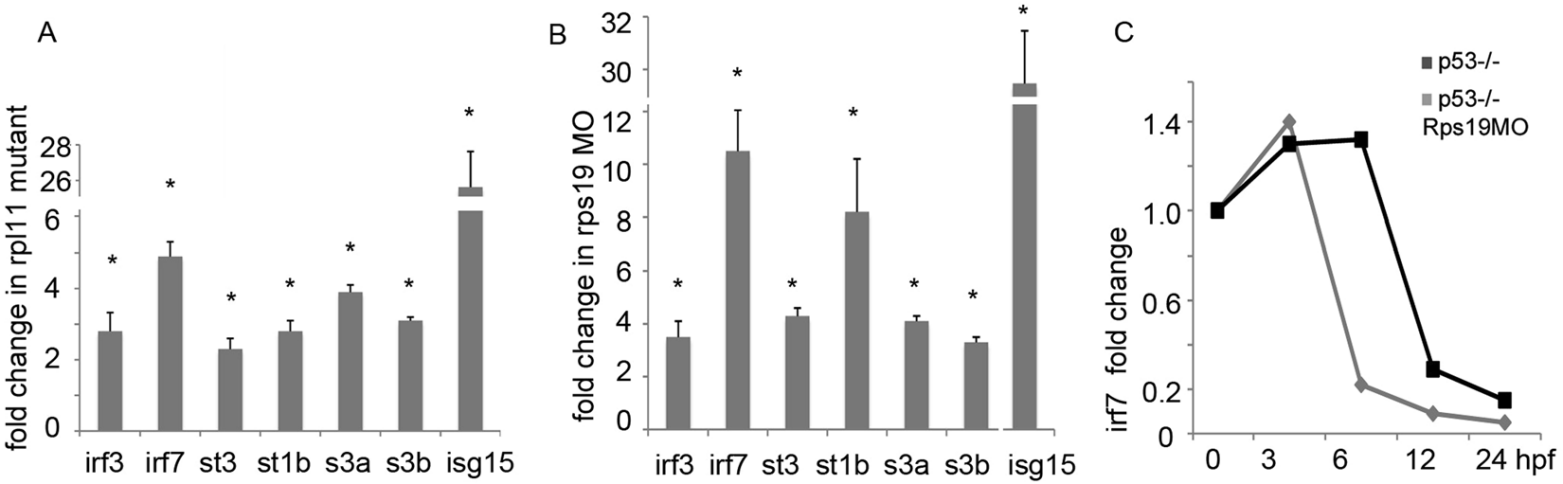
Interferon signaling was upregulated in RP-deficient zebrafish. (**A**) Interferon regulators *irf3* and *irf7* were upregulated in Rpl11 mutants along with signal transducers *stat3* and *stat1b* and interferon induced gene *isg15*, 48 hpf (hours post fertilization), RT-qPCR. Fold change in expression was calculated relative to wild-type embryos. RNA in this and other experiments was derived from a pool of at least 30 embryos. (**B**) The same set of genes was upregulated in embryos injected with Rps19 morpholino, 28 hpf, RT-qPCR. Injection of rps19 control mismatch morpholino did not upregulate genes from the interferon network. (**C**) *irf7* was upregulated in p53−/− mutant injected with Rps19 morpholino, RT-qPCR. (St1b, *stat1b*; st3, *stat3*; s3a, *socs3a*; s3b, socs3b. Bars represent the mean of three replicates ± sd. Asterisk indicates significant difference in comparison to controls, p < 0.05.

We examined expression of the components of the interferon network in RP-deficient zebrafish. Interferon regulatory factors Irf3 and Irf7 are key controllers of type I IFNs. They regulate the transcription of IFN-alpha and beta as well as transcription of IFN-stimulated genes (ISG) by binding to an interferon-stimulated response element in their promoters^46,47^. We found increased expression of both *irf3* and *irf7* in Rpl11 mutant and Rps19 morphants (Fig. 1A,B). INFs signal through STAT proteins to activate the transcription of interferon stimulated genes (ISGs). In RP-deficient zebrafish, *stat1b* and *stat3* were upregulated (Fig. 1A,B). IFN signaling induces several IFN inhibitors including *Socs3a and b* (suppressors of cytokine signaling 3) members of the STAT-induced STAT inhibitor family. These genes were also upregulated in both RP-deficient models (Fig. 1A,B).

Upregulation of ISG genes is another indicator of interferon system activation. The interferon-induced gene, *isg15* (ubiquiting-like modifier 15) is a sensitive indicator of interferon activation. The product of the *isg15* gene is an ubiquitin-like protein that is conjugated to intracellular target proteins upon activation by interferons^48^. *isg15* was the second-most upregulated gene in Rpl11 mutants according to microarray data verified by qPCR (Fig. 1A). It was also upregulated in Rps19-deficient zebrafish (Fig. 1B). A number of other ISG genes have been upregulated. They include a gene encoding Gbp2 (guanylate binding protein 2, interferon-inducible), which was 2.5 fold upregulated in Rpl11 mutant microarray and 2.8 fold upregulated in Rps19 morphants (data not shown). Gbp2 hydrolyzes GTP to GMP, promotes oxidative killing, and delivers antimicrobial peptides to autophagolysosomes^49^. An ISG gene encoding another GTP-metabolizing protein, MxA, was 2.2 fold upregulated in Rps19 morphants (data not shown). Mknk2b kinase that mediates suppressive effect of IFN-gamma on hematopoiesis was 2.5-fold upregulated in Rpl11 microarray. Another 3-fold upregulated ISG in Rpl11 mutant is *gtpbp1* (GTP binding protein 1) that promotes degradation of RNA species in exosomes. Therefore various components of interferon signaling network were upregulated in Rpl11 mutant and in Rps19 morphants.

There is cross talk between p53 and the interferon network. P53 has an IFN-stimulated response element in its promoter and therefore is induced by interferons^50^. In turn, p53 enhances interferon responses^51^. For that reason we examined the effect of p53 on the expression of the key IFN regulator *ifr7*. This gene was upregulated in *p53-/-* zebrafish mutant (Fig. 1C), which suggests that IFN network upregulation in RP-deficient zebrafish is at least partially p53 independent. The *irf7* expression decreases when development progresses, a pattern similar to *tp53* expression during development, which may be associated with decreasing stemness and increasing differentiation^52^.

### Expression of immune receptors signaling to IFN is altered in zebrafish models of DBA

In RP-deficient cells, interferons can be induced by endogenous ligands such as DNA and RNA from apoptotic bodies, non-processed faulty rRNA, and DNA fragments leaking to cytoplasm during DNA repair. RNA Pol III can convert them into RNA. Expression of several subunits of RNA Pol III have been upregulated in a microarray from Rpl11 mutants including *polr3f*, 2.3 fold, *polr3e*, 2.2 fold, *polr3k*, 2.1 fold, and a subunit of RNA Pol III transcription initiation factor *brf1*, 12 fold as has been confirmed by RT-qPCR. Therefore upregulated RNA Pol III could convert most DNA fragment originated during DNA repair into RNA, which can contain regions forming dsRNA.

**Figure 2:**
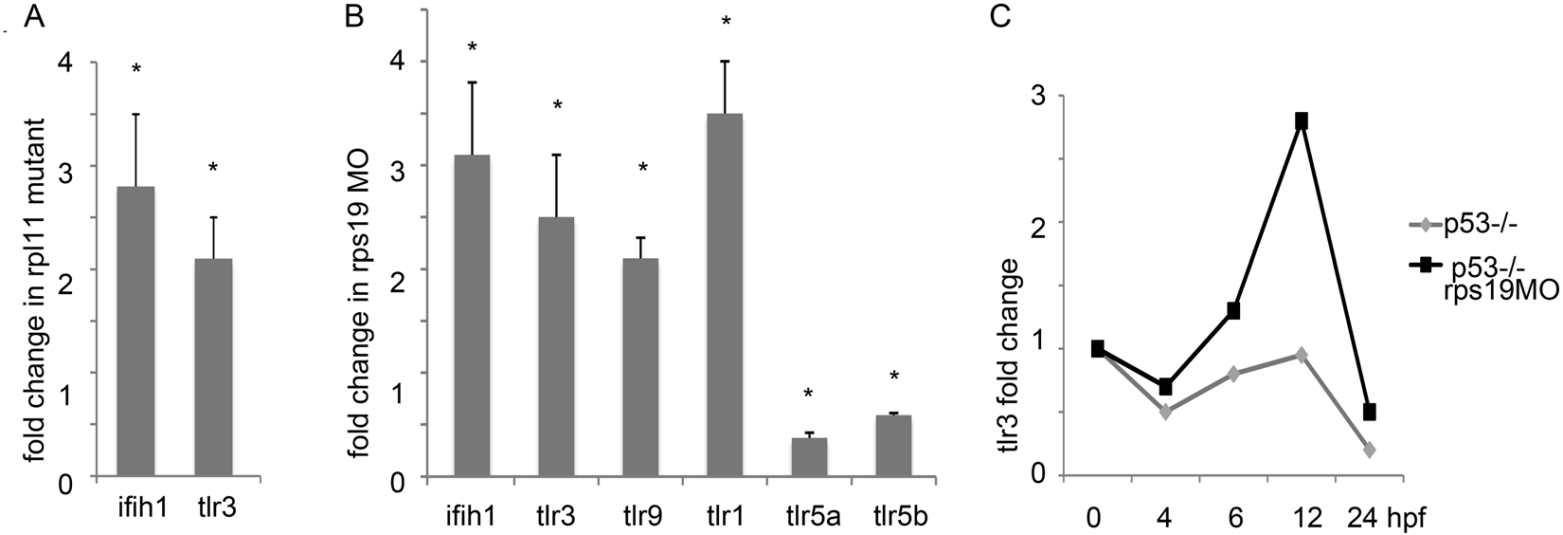
Expression of innate immune receptors was altered in RP-deficient zebrafish. (**A**) *ifih1 and tlr3* that recognize RNAs were upregulated in Rpl11 mutants. 48 hpf. RT-qPCR. Shown fold changes against wild type siblings (**B**) *ifih1* and *tlr3* were also upregulated in embryos injected with Rps19-specific morpholino in comparison to wild-type control. *tlr9* that recognizes DNA and *tlr1* that recognizes lipoproteins were also upregulated. Expression of *tlr5a/b* that recognize bacterial flagellin was decreased. 28 hpf, RT-qPCR,. (**C**) *tlr3* was upregulated in *tp53*−/− mutants injected with Rps19 specific morpholino. Fold change was calculated relative to tlr3 expression at 0 hpf. RT-qPCR. The bars represent the mean of three replicates ± sd. Asterisk indicates significant difference in comparison to controls, p < 0.05.

One of the major cytosolic sensors of dsRNA is an innate immune receptor Mda5 encoded by *ifih1* (interferon induced with helicase C domain 1) gene^32^. Mda5 signals through interferon regulatory factors Irf3 and Irf7 to induce type I interferons. *ifih1* that encodes Mda5, was upregulated both in Rpl11 mutants and in Rps19 morphants (Fig. 2A,B).

Toll-like receptor 3 is endosomal sensor of dsRNAs and other RNAs. RNA ligands can be delivered to endosomes through autophagy that is increased in DBA cells^53^. *tlr3* was upregulated both in Rpl11 mutants and in Rps19 morphants (Fig. 2A,B). Besides *tlr3*, we found upregulation of genes encoding toll-like receptor Tlr9, which recognizes DNA and Tlr1, which recognizes lipoproteins (Fig. 2B). No endogenous ligands have been identified for Tlr1 but literature suggests their existence since polymorphism in human TLR1 affects severity of allergy^54^. All TLRs, except for Tlr3, signal through MyD88 to induce NFkB and Irf7, while Tlr3 also induces Irf3-dependent transcription^55^.

Interestingly, the expression of genes encoding Tlr5α and Tlr5β, which recognize bacterial flagellin and do not have endogenous ligands was decreased (Fig. 2B). Furthermore Rpl11 mutant microarray data shows 4.4 fold decrease in level of defensin beta, 1.5 fold decrease in lysozymes, and 1.7 fold decrease in amyloid beta precursor. This suggests that at least some innate immune responses can be weakened in an RP-deficient organism.

It was shown that TLRs, especially TLR3, could be upregulated by p53 in response to DNA stressors^56,57^. To evaluate p53 contribution to *tlr3* upregulation we injected *tp53-/-* mutants with Rps19-specific morpholino and measured *tlr3* expression. *tlr3* was upregulated in a p53-negative background (Fig. 1C). This finding indicates that *tlr3* upregulation in RP-deficient zebrafish is at least partially p53-independent. Interestingly, *tlr3* expression decreases during development, a pattern also seen with *tp53* and *irf7*.

### Inflammatory pathways are upregulated in RP-deficient zebrafish

**Figure 3:**
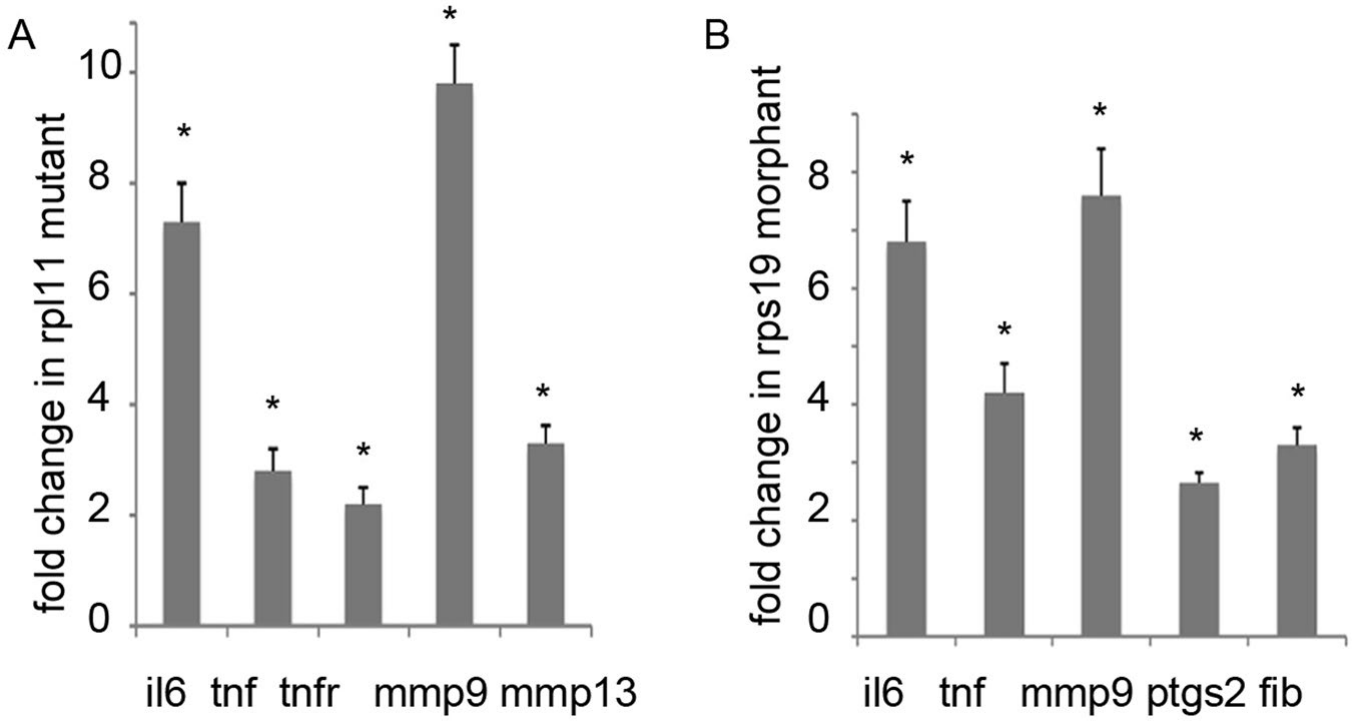
Pro-inflammatory genes and inflammatory mediators were upregulated in RP-deficient zebrafish. (**A**) In rpl11 mutant, expression of genes for cytokines Il6 and Tnf was increased along with upregulation of Tnf receptor and *matrix metallopeptidases 9 and 13*. Fold change in expression was calculated relative to wild-type siblings. 48hpf, RT-qPCR. (**B**) In rps19 morphants, *il6* and *tnf* were also upregulated along with *mmp9*, Cox2 enzyme encoding *ptgs2*, and fibrinogen. 24hpf, RT-qPCR. Fold change in expression was calculated relative to wild-type embryos. Bars represent the mean of three replicates ± sd. tnfr, *tnfrsf1a*, fib, *fibrinogen.*

Upregulation of innate immune receptors may lead to the activation of inflammatory pathways. We found upregulation of several pro-inflammatory cytokines and inflammatory mediators in RP-deficient zebrafish embryos (Fig. 3A,B). This includes cytokines interleukin 6 (*il6*) and tumor necrosis factor (*tnf*) along with its receptor *tnfrsf1a*, *matrix metallopeptidases 9 and 13*, fibrinogen, and *ptgs2* (prostaglandin-endoperoxide synthase-2), encoding for the Cox-2 enzyme. Cox-2 catalyzes the rate-limiting step of prostaglandin biosynthesis and is the target of non-steroidal anti-inflammatory drugs like aspirin.

### TGFβ signaling is altered in Rps19-deficient zebrafish and in RPS19-deficient human cells

**Figure 4:**
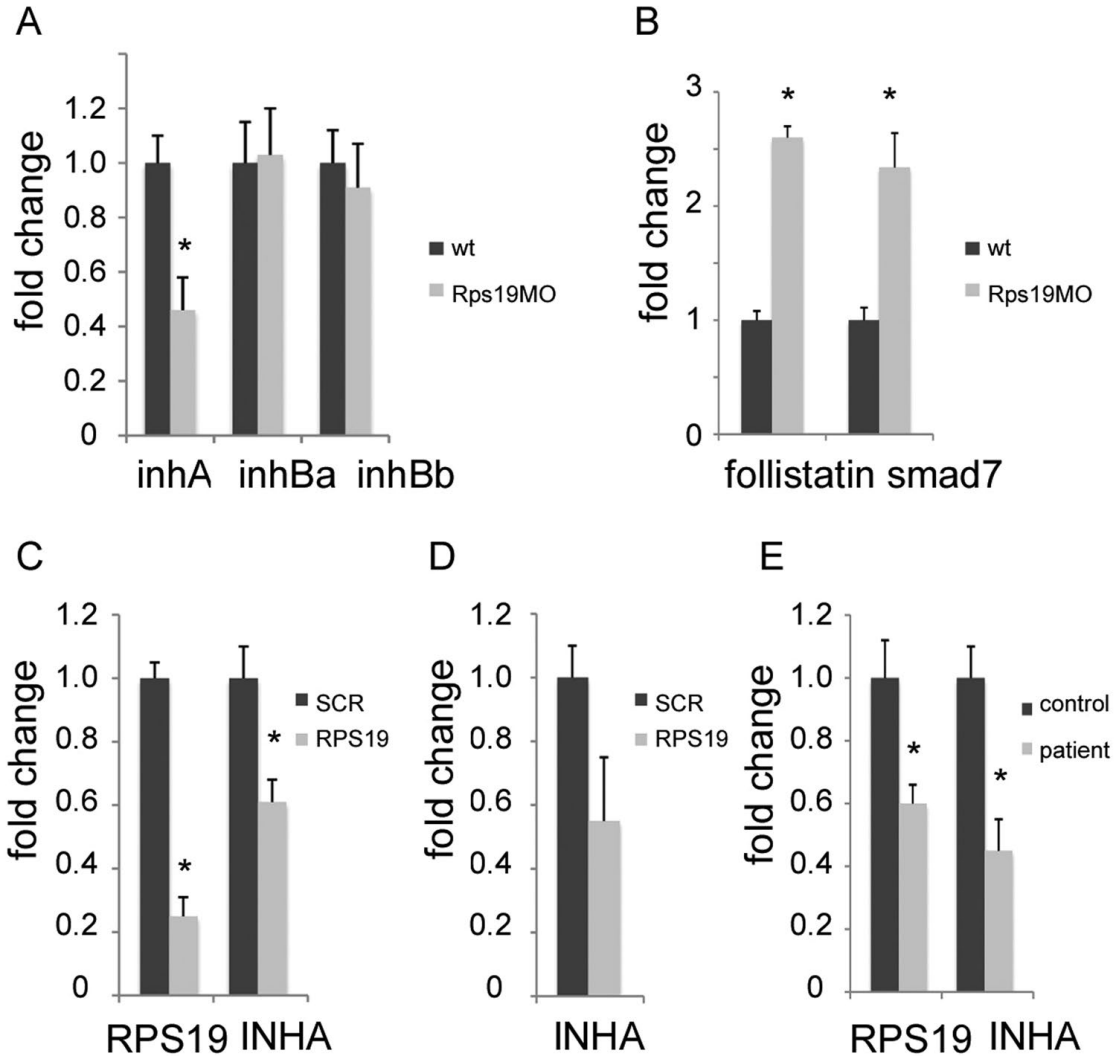
Expression of activin/inhibin was altered in RP-deficient zebrafish and human cells. (**A**) *inhA*, encoding a subunit unique to inhibin, was downregulated in Rps19-deficient zebrafish embryos while expression of genes for subunits common to both inhibin and activin was not changed, 40 hpf, RT-qPCR. (**B**) Follistatin, which is an activin inhibitor, was upregulated in Rps19-deficient zebrafish along with upregulation of inhibitory Smad7, 40 hpf, RT-qPCR. (**C**) K562 cells transduced with anti-RPS19 shRNA showed decreased expression of RPS19 and INHA subunit, SCR, scrambled shRNA control, RT-qPCR. (**D**) Inhibin alpha was also downregulated in human fetal liver cells deficient in RPS19, SCR, scrambled shRNA control, RT-qPCR. (**E**) Inhibin alpha was downregulated in a lymphoid cell line derived from a DBA patient with a mutation in the RPS19 gene, RT-qPCR. Bars represent the mean of three replicates ± sd. Asterisk indicates significant difference in comparison to controls, p < 0.05.

Toll-like receptor agonists can stimulate release from macrophages of activin A, a pro-inflammatory cytokine from the TGFβ family^58^. We therefore examined expression of activin subunits in RP-deficient zebrafish. Activin is counterbalanced by inhibin, a factor with an opposite activity. Both activin and inhibin are dimers. Activin is composed of two beta subunits that can be identical or different. Inhibin shares beta subunits with the activin, but the other subunit, alpha, is unique to inhibin. Therefore, changes in expression of the alpha subunit would affect the amount of beta subunits available for inhibin and, consequently, affect the activin/inhibin ratio. In Rps19 deficient zebrafish, expression of a subunit InhAa, unique to inhibin, was decreased, while expression of subunits inhBa and inhBb common for both inhibin and activin, was unchanged (Fig. 4A). It means the activin/inhibin equilibrium was shifted to activin in RP-deficient zebrafish. Another indicator of activin overproduction was upregulation of follistatin, a protein that binds and inhibits activin (Fig. 4B). We also found upregulation of *smad7*, an inhibitory SMAD that suppresses TGFβ signaling.

Inhibin alpha (*INHA*) was also among downregulated genes in cellular models of DBA. We transduced human human CD34+ fetal liver cells and K562 cells (*tp53*-/-) with lentiviral vectors expressing short hairpin RNA (shRNA) against *RPS19*^43,59^. In this system, we achieved ~50% reduction in RPS19 protein level^34,50^. In K562 cells with RPS19 knockdown, microarray analysis revealed *INHA* among the most downregulated genes (Fig. 4C). Since no changes in expression of beta subunits *INHBA* and *INHBB* have been detected by microarray, these data point to activin overproduction in these cells. *INHA* expression was also decreased in RPS19-deficient human fetal liver (Fig. 4D), and in lymphoid cell lines from a DBA patient with an RPS19 mutation (Fig. 4E).

These data suggest TGFβ family member activin with proinflammatory properties was over-produced in zebrafish DBA models and in human cells deficient in RPS19. Activin overproduction was detected both in a wild type and a p53-negative background. This suggests that the increase in activin production in DBA models does not depend on p53.

### Complement system is upregulated in RP-deficient zebrafish

Complement system can be activated by different mechanisms even in absence of infection. Spontaneous C3 hydrolysis and binding of C3 convertase enzyme to cells initiate the alternative pathway of complement^26^. Normally, it is limited by activity of factor H and complement regulatory proteins acting on cell surface to clear complement complexes. In pathological conditions the balance between complement activation and inhibition is broken and C3 convertase can recruit more complement proteins onto the cell surface leading to the formation of the membrane attack complex, cell damage, activation of phagocytosis, and inflammation.

**Figure 5:**
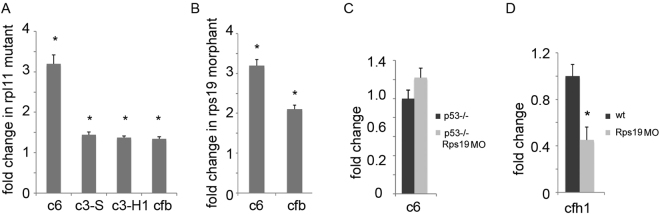
Complement system was upregulated in RP-deficient zebrafish. (**A**) Complement components *c6*, *c3-S*, *c3-H1*, and *cfb* were upregulated in Rpl11 mutant zebrafish embryos, 48 hpf, RT-qPCR. (**B**) Complement components *c6* and *cfb* were upregulated in Rps19-deficient zebrafish. 24 hpf, RT-qPCR (**C**) On *tp53*-/- background, *c6* upregulation was much smaller, 24 hpf, RT-qPCR. (**D**) Inhibitor of complement factor H was downregulated in Rps19-deficient zebrafish embryos, 24 hpf, RT-qPCR. Bars represent the mean of three replicates ± sd. Asterisk indicates significant difference in comparison to controls, p < 0.05.

In Rpl11 microarray confirmed by RT-qPCR we found significant upregulation of complement component 6 (Fig. 5A). C6 is a part of the membrane attack complex. C3-S and C3-H1 isoforms of complement component 3 and complement factor B (*cfb*) from the alternative complement pathway were also somewhat upregulated in Rpl11 mutants (Fig. 5A). In Rs19-deficient zebrafish, complement component C6 was also upregulated along with an increase in the expression of factor B (Fig. 5B). On *tp53*-/- background upregulation was smaller (Fig. 5C) suggesting p53 activation contributes to complement activation. While complement components were upregulated, expression of complement inhibitor factor H was decreased (Fig. 5D). RNA-seq of Rps19-deficient zebrafish also suggested upregulation of c6 complement component and downregulation of inhibitory factor H^60^.

### Application of inhibitors of TLR3 receptor, activin receptor, and complement improved the condition of RP-deficient zebrafish

**Figure 6:**
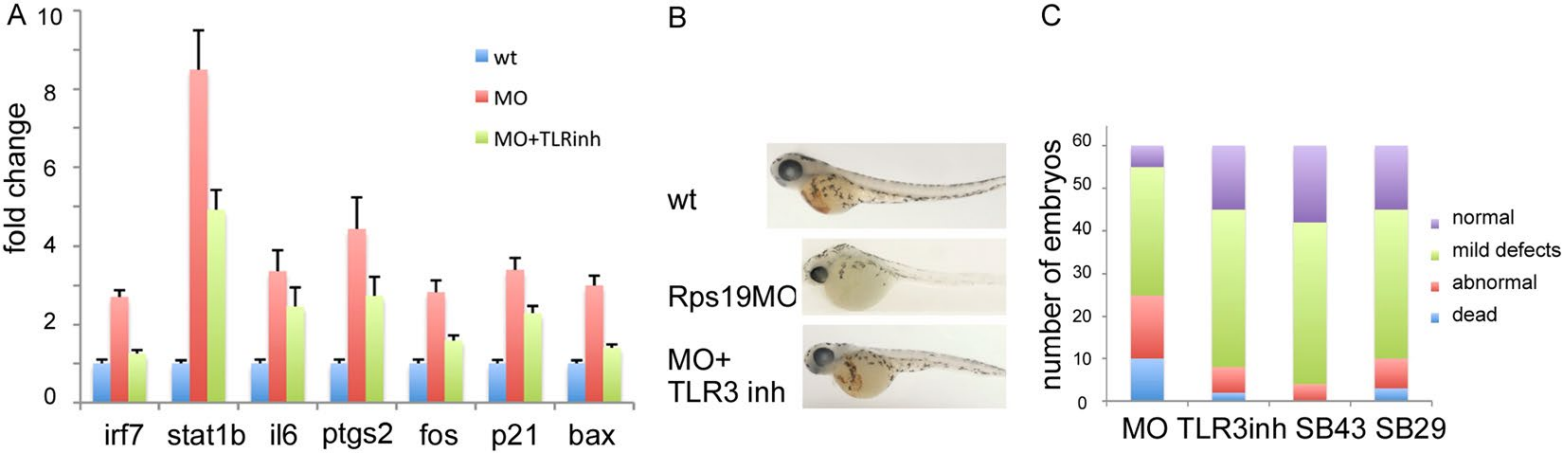
Inhibitors of TLR3, activin, and complement partially rescued Rps19-deficient zebrafish embryos. (**A**) TLR3 inhibitor decreased expression of interferon mediators *irf7* and *stat1b*, decreased expression of inflammatory markers *il6* and *ptgs2/cox2* and acute phase response gene *fos*. It also downregulated *p21* responsible for cell cycle arrest and pro-apoptotic inflammatory gene *bax*, 40 hpf, RT-qPCR. Bars represent the mean of three replicates ± sd. Asterisk indicates significant difference in comparison to controls, p < 0.05. (**B**) TLR3 inhibitor improved hematopoiesis in Rps19 morphants, day 3, o-dianizidine staining. 50 embryos per group, representative staining is shown. (**C**) Embryos were injected with rps19 morpholino, MO group was left untreated, other groups were treated with TLR3 inhibitor (TLRinh), SB431542 activin inhibitor (SB43) or SB290157 complement inhibitor (SB29). Treatments decreased morphological defects and improved survival in Rps19-deficient zebrafish embryos. Embryos classified as abnormal had strong defects such as tail kinks, curved body, strongly shortened body, arrest at early developmental stage, and underdeveloped eyes. Embryos with mild defects had smaller size, smaller eyes and heads. 24 hpf. The experiment was performed in triplicates, 60 embryos per group; bars represent the mean of three replicates. Number of dead/abnormal/mild/normal embryos for MO 10 ± 0.6/15 ± 0.6/30 ± 1.2/5 ± 1; for TLR3 inhibitor 2 ± 1/6 ± 1/37 ± 1/15 ± 0.6; for SB431542 inhibitor 0 ± 0/4 ± 1/38 ± 2.5/18 ± 1.5; for SB2990157 3 ± 1/7 ± 0.5/35 ± 1.2/15 ± 0.7.

Our data suggested that upregulation of Mda5 and some TLRs leads to activation of interferons, inflammation, activin, and complement, which might contribute to the RP-deficient phenotype. We therefore examined effects of inhibitors of these pathways on RP-deficient zebrafish. We injected zebrafish embryos with Rps19-specific morpholino and treated them with TLR3 receptor inhibitor, compound SB431542 that inhibits activin receptor, or compound SB290157 that acts as a competitive antagonist of anaphyloxin C3a receptor. Application of TLR3 inhibitor resulted in downregulation of interferon mediators *irf7* and *stat1b*, suggesting decrease of interferon system activation (Fig. 6A). Expression of pro-inflammatory markers *il6* and ptgs2/*sox2* was also decreased after treatment as well as expression of acute-phase response gene *fo*s. We also observed downregulation of *p21* responsible for cell cycle arrest and a pro-apoptotic gene *bax*. As a result, we observed partial rescue of hematopoiesis (Fig. 6B) and morphology (Fig. 6C) in Rps19-deficient embryos. Inhibitors of activin and complement also improved hematopoiesis and morphology of Rps19-deficient embryos.

## Discussion

**Figure 7:**
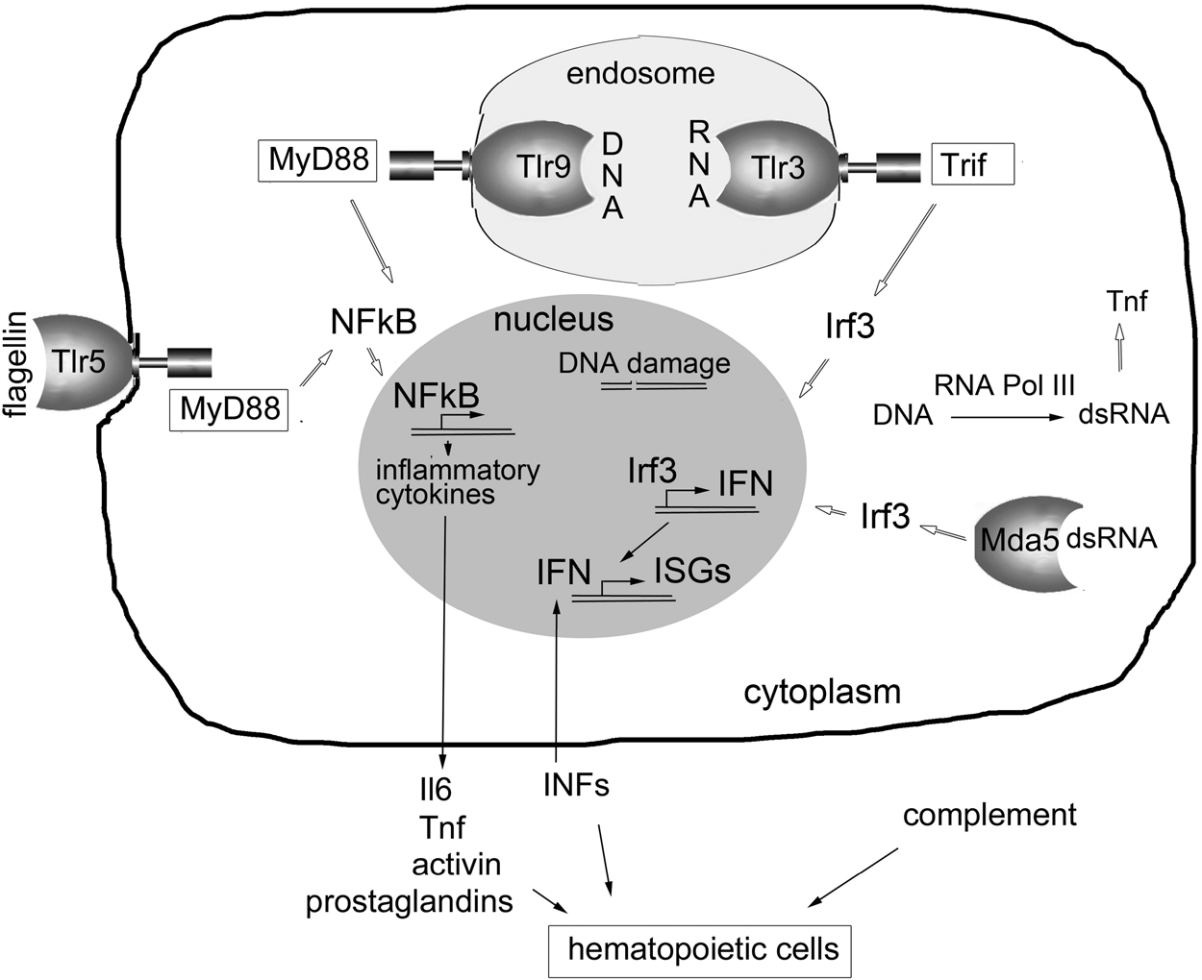
Response of innate immune system to RP-deficiency. Tlr3 and Tlr9 receptors are expressed in endosomes and can be activated by endogenous RNAs and DNAs that comes from apoptotic cells and autophagosomes. In addition, cytoplasmic sensor of dsRNAs Mda5 (encoded by *ifih1*) was upregulated in RP-deficient zebrafish. During DNA damage repair DNA fragments can escape into cytoplasm and converted by RNA Pol III into RNAs that may contain dsRNA regions. Once activated, receptors induce expression of interferons and pro-inflammatory cytokines, which are released and act on hematopoietic cells. Several factors may synergize in decreasing proliferation of hematopoietic cells and shortening their life span.

In RP-deficient zebrafish, we found upregulation of interferons, inflammation (TNF, il6), activin, and complement. Many changes in RPs-deficient cells may contribute to the activation of these immune mechanisms. They include accumulation of defective pre-rRNAs, DNA damage, increased ROS, increased apoptosis, alterations in composition of membranes and extracellular matrix and metabolic defects among major causes. RNA and RNA from apoptotic bodies or from autophagosomes can get into endosomes where they are recognized correspondingly by Tlr3 and Tlr9 (Fig. 7). Both these receptors have been upregulated in RP-deficient zebrafish.

We also found upregulation of cytosolic receptor Mda5 that recognizes mostly dsRNA. Upregulation of this receptor can be related to DNA damage we previously found in DBA models^43^. Induction of interferon by DNA damage caused by radiotherapy and chemotherapy drugs is well known and the mechanism of IFN induction was traced to DNA fragments that are generated during DNA double strand break repair^38^. Some of them can migrate to the cytosol where they are recognized by DNA sensors or are converted into dsRNA by RNA PolIII and recognized by RNA sensors such as Rig1 and Mda5^40^. To prevent immune activation, DNA fragments are destroyed by 3′–5′ exonuclease Trex1 located on the outer side of the nuclear membrane. Deficiency of this enzyme as well as malfunction or insufficient activity of DNA repair proteins can cause accumulation of DNA fragments in the cytoplasm and stimulate NF-kB and IFN pathways^38,39^. In addition, delayed processing of nascent pre-rRNA could interfere with transcription and increase the production of DNA-RNA hybrids or their delayed removal, which would result in activation of innate immune sensors and IFN induction^61^.

Activation of the complement system in RP-deficient zebrafish may be caused by accumulation of apoptotic cells^41^ and by the alterations in composition of membranes that have been reported in DBA^42^. Our data suggest increased activity of the alternative complement pathway in our models. In this pathway, C3 is cleaved by a complex of C3b with Bb serine protease (C3bBb convertase)^26^. In an auto amplification loop, C3b generated by this complex, binds to a proenzyme complement factor B leading to its cleavage by factor D and generation of more C3bBb. Complement factor H competes with Bb for binding to C3b promoting the decay of the convertase. It also serves as a cofactor for complement factor I that cleaves C3b. Erythrocytes express several complement regulators and receptors. Specifically, erythrocytes maturing in association with macrophages express several C3b decay factors and C3b-specific complement receptor CR1, which expression gradually decreases during maturation^62^. This suggests early erythrocytes are sensitive to complement. In RP-deficient zebrafish, expression of factor B was increased while that of factor H was decreased, which may increase 3b deposition on erythrocytes promoting their engulfment by macrophages and would decrease a proportion of erythrocytes that are able to mature. In addition, we found increased expression of complement factor 6. C6 is a key member of the membrane attack complex that induces cell lysis. Although lysis of erythrocytes is not a prominent feature of DBA, it may take place at the low level. Our data suggest DBA may join the growing list of diseases with over activated complement.

Both interferons and complement system can promote inflammation. In RP-deficient zebrafish we found upregulation of inflammatory cytokines and mediators. We also found decreased expression of inhibin-specific subunits in RP-deficient zebrafish and human cells, which suggest a shift to activin in the inhibin-activin balance. Upregulation of IFNs inhibits hematopoiesis^63^. Inflammatory cytokines such as TNF and activin can also suppress hematopoiesis^64^.

In adult fish and in human patients, which have both innate and adaptive immune systems, activation of innate immune receptors may affect T and B cells. For example, upregulation of interferons may activate macrophages and direct T helper cell development to the pro-inflammatory Th1 pathway^65^. Increased proportion of cytotoxic T cells (decreased T4/T8 ratio) was reported in DBA and may also be connected to interferon activation^15^. Interferons may contribute to the apoptosis of hematopoietic progenitors induced by cytotoxic T cells in acquired aplastic anemia^66^.

Conflicting results associated with studies of immunity in DBA may be explained by differences in status of the analyzed patients. Many patients are on corticosteroid therapy, which suppresses inflammation. In patients not treated by corticosteroids, autonomous immune response may vary depending if they are in an acute phase or in remission, which would be associated with variation in p53 activity. P53 suppresses NFkB and NFkB-mediated inflammation^67^. P53 however promotes interferon responses^51^.

Our data suggest that the right balance between p53 and immunity is necessary to promote cell health. Glucocorticoids used in DBA treatment may work in part by suppressing inappropriate immune activation. Inhibitor of TLR3 receptor, inhibitor of activin receptor, and complement inhibitor also improved the condition of RP-deficient embryos. There are other examples of application of these inhibitors. Blockade of TLR3 was recently shown to protect mice from lethal radiation induced syndrome^68^. Cytokine inhibition has been successfully used to treat some rheumatic and autoimmune diseases^69^ and antibodies targeting interferons and interleukins are being investigated for SLE. Sotatercept that suppresses activin signaling is investigated as a potential therapeutic for anemia^70^. Overall our data suggest that innate immune pathways may contribute to the pathogenesis of DBA. Based on our findings, therapies targeting innate immune response or modulating activin signaling should further be explored as potential treatments for DBA.

## Materials and Methods

### Zebrafish

Zebrafish (*Danio rerio*) lines used AB, *rpl11*^*hi3820bTg*^ ^45^ and *tp53*^*zdf1/zdf1*^ ^71^. Embryos were obtained by natural spawning. UCLA Animal Committee approved the study and all methods were performed in accordance with the relevant guidelines and regulations.

### Human primary cells and cell lines

EBV-immortalized lymphoid cell line from a DBA patient with an RPS19 mutation and a cell line from a normal control were a gift from Hanna Gazda (Harvard University, Boston, MA). Both cell lines were grown in RPMI-1640, supplemented with 10% FBS. Human fetal liver tissues were obtained from Advanced Bioscience Resources Inc (Alameda, CA). Cells were sorted for CD34^+^ using MACS cell separation (Miltenyi Biotec, Auburn, CA). Sorted cells were grown in x-Vivo15 media (Lonza, Basel, Switzerland) containing 10% FBS, 50 ng/mL of FLT-3, TPO, and SCF and 20 ng/mL of IL-3 and IL-6. K562 cells were grown in RPMI-1640, supplemented with 10% FBS. Primary CD34+ fetal liver cells and K562 cells were transduced with lentivirus containing shRNA against RPS19 or scrambled (SCR) shRNA, sorted for GFP at 72 hours, and harvested post-transduction, as indicated in results.

### Microarray

K562 cells were transduced with lentivirus carrying shRNA against RPS19 or control scrambled shRNA and sorted for the GFP marker. RNA was purified with Trizol and analyzed by microarray on an Affymetryx platform. The microarray and data analysis were performed at the UCLA DNA Microarray Core. Preparation of microarray of Rpl11 mutant has been reported previously^12^.

### RT-qPCR

RNA was prepared using Trizol (Invitrogen, Carlsbad, CA) from 30–40 embryos or from 1–5 million cells; 2 μg was used for RT with the random hexamer primers. PCR was performed in triplicates using iQ SYBR Green Super Mix. Primers are shown in a Supplemental Table 1. Levels of mRNA were normalized to beta-actin and calculated by Cτ method.

### Morpholinos

3 ng of the following morpholinos were injected at the one cell stage: Rps19–specific morpholino targeting exon 3 splicing site 5′- gcttccccgacctttcaaaagacaa and 5 bases mismatch: 5′- gattcctcgaactctcaatagacaa (Gene Tools, Philomath, OR).

### Staining of erythroid cells

6 mg of o-dianizidine was dissolved in 50 μl of acetic acid, diluted to 6 ml by water, and neutralized by 10 μl of 10 M NaOH following by 4 ml of ethanol and 130 μl of 50% H_2_O_2_. Embryos were placed in this solution and color development was monitored under a microscope. Embryos were washed with water before imaging.

### Drug treatments

100 mM stocks of TLR3 receptor inhibitor (Calbiochem), compound SB431542 (Tocris Bioscience, Minneapolis, MN), and compound SB290157 (Santa Cruz Biotechnology, CA) were prepared in DMSO and added to fish water to 3 μM concentrations.

### Statistics

Each experiment was repeated at least twice, and results of a representative experiment are shown. Data are presented as the means of at least tree measurements plus/minus SD. Student’s *t* test was used for comparisons of a variable between two groups.

### Data availability

Microarray of Rpl11 mutant data are available at the depository ArrayExpress, Acc: E-MEXP-2381.

## Electronic supplementary material

Dataset 1
