# Supplementary material for: Innate immune system activation in zebrafish and cellular models of Diamond Blackfan Anemia
Source: Sci Rep. 2018 Mar 26;8:5165. doi: 10.1038/s41598-018-23561-6 (PMC5980095; doi:10.1038/s41598-018-23561-6)
Supplement: Supplementary file 1 — Dataset 1 [file 41598_2018_23561_MOESM1_ESM.docx]

**Innate immune system activation in zebrafish and cellular models of**

**Diamond Blackfan Anemia**

Nadia Danilova,^1^ Mark Wilkes,^2^ Elena Bibikova,^2^ Min-Young Youn,^2^ Kathleen M. Sakamoto,^2*^ and Shuo Lin^1*^

^1^Department of Molecular, Cell & Developmental Biology, University of California, Los Angeles CA; ^2^Department of Pediatrics Stanford University School of Medicine, Stanford, CA, USA

**Supplemental Data**

**Supplemental Table 1**. **Primers used in qPCR.**

| Gene | Name | Sequence |
| --- | --- | --- |
| Zebrafish:  actin beta  bax  brf1  complement factor B  complement factor 6  complement factor H  fibrinogen  follistatin  fos  gbp2  ifih1  interleukin 6  inhibin A  inhibin Ba  inhibin Bb  irf3  irf7  isg15  mmp9  mmp13  mxA  ptgs2/cox2  p21  smad 7  socs3a  socs3b  stat1b  stat3  tlr1  tlr3  tlr5a  tlr5b  tlr9  tnf  tnfrsf1a  Human:  Actin  RPS19  INHBA | ac  acr  bx  bxr  brf1  brf1r  cfb  cfbr  cf6  cf6r  cfh1  cfh1r  fib  fibr  fol  folr  fos  fosr1  gbp2  gbp2r  ifih1  ifih1r  il6  il6r  inha  inhar  inhBa  inhBar  inhBb  inhBbr  irf3  irf3r  irf7  irf7r  isg15  isg15r  mmp9  mmp9r  mmp13  mmp13r  MxA  MxAr  cox2  cox2r  z21  z21r  sm7  sm7r  socs3a  socs3ar  sos3b  socs3br  stat1b  stat1br  stat3f  stat3r  tlr1  tlr1r  tlr3  tlr3r  tlr5a  tlr5ar  tlr5b  tlr5br  tlr9  tlr9r  TNFa  TNFar  tnfr  tnfrr  F  R  F  R  F  R | 5’-TCTCTTCCAGCCTTCCTTCCT  5’-CTCATCGTACTCCTGCTTGCT  5’-CGTCGGGTGGAGGCGATACG  5’-GAGTCGGCTGAAGATTAGAGTT  5’- CCTTTGTGGAGAGTGGCGGC  5’-GACGAGTGCTGATTCATCTGAA  5’- AAAGGAATCGAAGTGGCAGA  5’- TGCATCAAGTTTCGCTTTTG  5’-AACCGAGCATTCCAAAGGAC  5’-AACGGTCAGCATGAAAAGCA  5’-TGACGCTCCACCAAAAGTTG  5’-CCTGGGACACTTTGCTTCAC  5’- CAGCAGTCCCCCTCTTACTG  5’-CATGACACCTGGATGCAAAC  5’- ATTACTTTTGCGCTGCTCGT  5’- TCCCAGGCATGTAGAGAACC  5’-TACCAGCCTTAACGCCGACT  5’-GCTTCTCTTGTTGGAGGTCTT  5’- GCAGGAGAAAGAGGTGCAGA  5’- CGAGGGCTTAGACTCCTCCT  5’-GAGCCGCCGTCTAAAATCAG  5’-AATGACTCCGTTGGTCTCGT  5’-TCCTGGTGAACGACATCAAA  5’-TCATCACGCTGGAGAAGTTG    5’-CCTTGAAGGTTTGGGGTTGG  5’-CCAGGTCCAGCATCAGAAGA  5’-ACGCCATCCGTAAGGTACAC  5’-GCAGTCGAAGGAAGATCCAG  5’-TTCACGCGGGTAAAGTTAGG  5’-TTTGCCTGCAACACGTAGAG  5’-GCCGAGGTCGATCTCAATAA  5’-ACTGCAGTGAAGACGCTCCT  5’-TGCAACAGAACTCCAAGGAA  5’-ACTGAAGGCAGACCCAGAGA  5’-AAAAATCAGCGAAAGCCTCA  5’-TGGGCACGTTGAAGTACTGA  5’- AGGCAAGGTGCTCCTGTTTA  5’- ACTGCATTTCAGGAGGTCGT  5’-AGGAGACCAAGACACACTCG  5’- TTTCATCACCTCCAGCGTCT  5’-GTCAGGGACCAGATCAAGCT  5’-GGCTGTAAACGATGAGCTCC  5’-CATTCGCAACATGGTGGACT  5’-TGACCGTACAGCTCCTTCAG  5′-TGAGAACTTACTGGCAGCTTCA  5′-AGCTGCATTCGTCTCGTAGC  5’-CCCCTATGGGGTTTTCAGAT  5’-GTGCCCTGAGGTAGGTCGTA  5’- GGAAGACAAGAGCCGAGACT  5’- CCTTGGAGCTGAAGGTCTTG  5’-TTGCTCAAGGGGTTTTATGG  5’-TTGCTCAAGGGGTTTTATGG  5’-ACACACTTGCTGCTCCAATG  5’-AAACCTTGCAACGGGTCTTG  5’-GACACTGAGTTCCCTGACACA  5’-CTTCTCTCATGGGTGACGCAT  5’-TCCTGCAGACATCCACACTT  5’-CAGAGAGGCAAAATCACGCA  5’-GGTGACCCTGGTTCTCTCTG  5’-TGCTGCTATCCAACACCAAG  5’- GAGAACAAGAGAAACCCGCG  5’- TTTGGCACCTCTTGAAGACC  5’- TGGGAGTAATGGTCTGCGTT  5’- TCCCATTCTGTGAGCACTGT  5’-TCAGAGTTGGATTGCAAACGT  5’-GAGAAGTGAACCTGGGGACT  5’-GGTGTTTGGGATCATTTTGG  5’-CAAGCCACCTGAAGAAAAGG  5’- TGCACAAAACCCCATTCCAG  5’- ATCGCACAGCTTACAGGAGT  5’- CCATTGGCAATGAGCGGTT 5’- GCGCTCAGGAGGAGCAA   \| 5’- GCCTGGAGTTACTGTAAAAGACG 5’- CCCATAGATCTTGGTCATGGAGC \| \| --- \|   5’AAGTCGGGGAGAACGGGTATGTGG 5’-TCTTCCTGGCTGTTCCTGACTCG |
